# Supplementary material for: Angular-spectrum-dependent interference
Source: Light Sci Appl. 2021 Oct 26;10:217. doi: 10.1038/s41377-021-00661-z (PMC8548309; doi:10.1038/s41377-021-00661-z)
Supplement: Supplementary file 1 — Supplementary Materials [file 41377_2021_661_MOESM1_ESM.docx]

Supplementary Information for

Angular-spectrum-dependent interference

Chen Yang^1,2^, Zhi-Yuan Zhou^1,2^*, Yan Li^1,2^, Shi-Kai Liu^1,2^, Zheng Ge^1,2^, Guang-Can Guo^1,2^ and Bao-Sen Shi ^1,2^*

^1^ CAS Key Laboratory of Quantum Information, University of Science and Technology of China, Hefei, Anhui 230026, China

^2^ Synergetic Innovation Center of Quantum Information & Quantum Physics, University of Science and Technology of China, Hefei, Anhui 230026, China

[* Correspondence: (Zhi-Yuan Zhou) *zyzhouphy@ustc.edu.c*n or (Bao-Sen Shi) *drshi@ustc.edu.cn*](mailto:*%20e-mail:%20zyzhouphy@ustc.edu.cn;%20drshi@ustc.edu.cn)

1. The quasi-phase-matching conditions


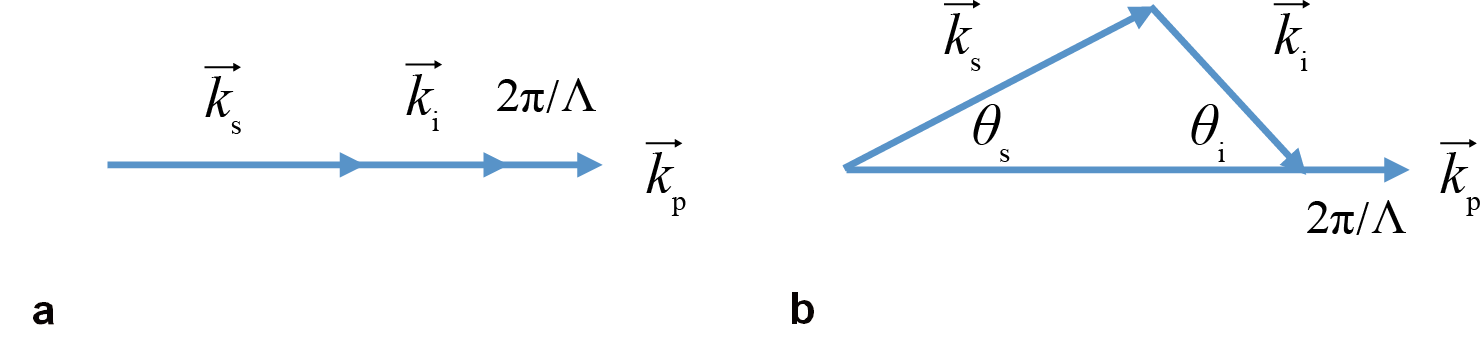


Fig. S1 | Geometric relation among the wavevectors in SPDC. **a** the collinear case; **b** the non-collinear case.

In general, the phase mismatch of quasi-phase-matching is given by ^1^

$\Delta k=k_{\text{p}}-k_{\text{s}}-k_{\text{i}}-\frac{2\pi}{\Lambda}$ (S1)

where $k_{j}=n\left(\omega_{j}\right)\frac{\omega_{j}}{c}, j=\text{s},\text{i},\text{p}$;$\omega, c, n$ represent angular frequency, the speed of light, and refractive index respectively; $\Lambda$ represents the grating period of the crystal. The geometry of the wavevectors is shown in Fig. S1a. We here are interested in the non-collinear phase matching case. As shown in Fig. S1b, three wavevectors $k_{\text{p}}-2\pi/\Lambda$, $k_{\text{i}}$, and $k_{\text{s}}$ form a triangle. From the geometry, one can write the mismatch as

$\Delta k=k_{\text{p}}-k_{\text{s}}\cos\theta_{\text{s}}-k_{\text{i}}\cos\theta_{\text{i}}-\frac{2\pi}{\Lambda}$ (S2)

When we consider the conservation of transverse momentum $k_{\text{s}}\sin\theta_{\text{s}}=k_{\text{i}}\sin\theta_{\text{i}}$ and the law of refraction$\sin\theta_{\text{s\_out}}=n\left(\omega_{\text{s}}\right)\sin\theta_{\text{s}}$, where $\theta_{\text{s}}$ is the angle in the crystal, $\theta_{\text{s\_out}}$ is the outside angle, and in a small-angle approximation the refractive index $n\left(\omega\right)$ is only dependent on wavelength and independent of angle, then the mismatch can be written as

$\Delta k=k_{\text{p}}-\sqrt{k_{\text{s}}^{2}-k_{\text{s}}^{2}\frac{\sin^{2}\theta_{\text{s\_out}}}{n^{2}\left(\omega_{\text{s}}\right)}}-\sqrt{k_{\text{i}}^{2}-k_{\text{s}}^{2}\frac{\sin^{2}\theta_{\text{s\_out}}}{n^{2}\left(\omega_{\text{s}}\right)}}-\frac{2\pi}{\Lambda}$ (S3)

1. Derivations from the FAS to the interference pattern

The photon number generated is proportional to a $\text{sinc}^{2}$ function ^2^

$C_{1}\left(\lambda_{\text{s}},\theta_{\text{s\_out}}\right)=\alpha\text{sinc}^{2}\left(\frac{\Delta k\cdot L}{2}\right)$ (S4)

where $L$ represents the length of crystal, $\alpha$is a normalized factor, $\Delta k$is given by Eq. (S3). Now, one can obtain the frequency-angular spectrum (FAS) in Fig. S2a (the same as that in Fig. 1a in the main text) by calculating and normalizing Eq. (S4). The Sellmeier equation that we use to obtain the refractive index is from ref. ^3^.


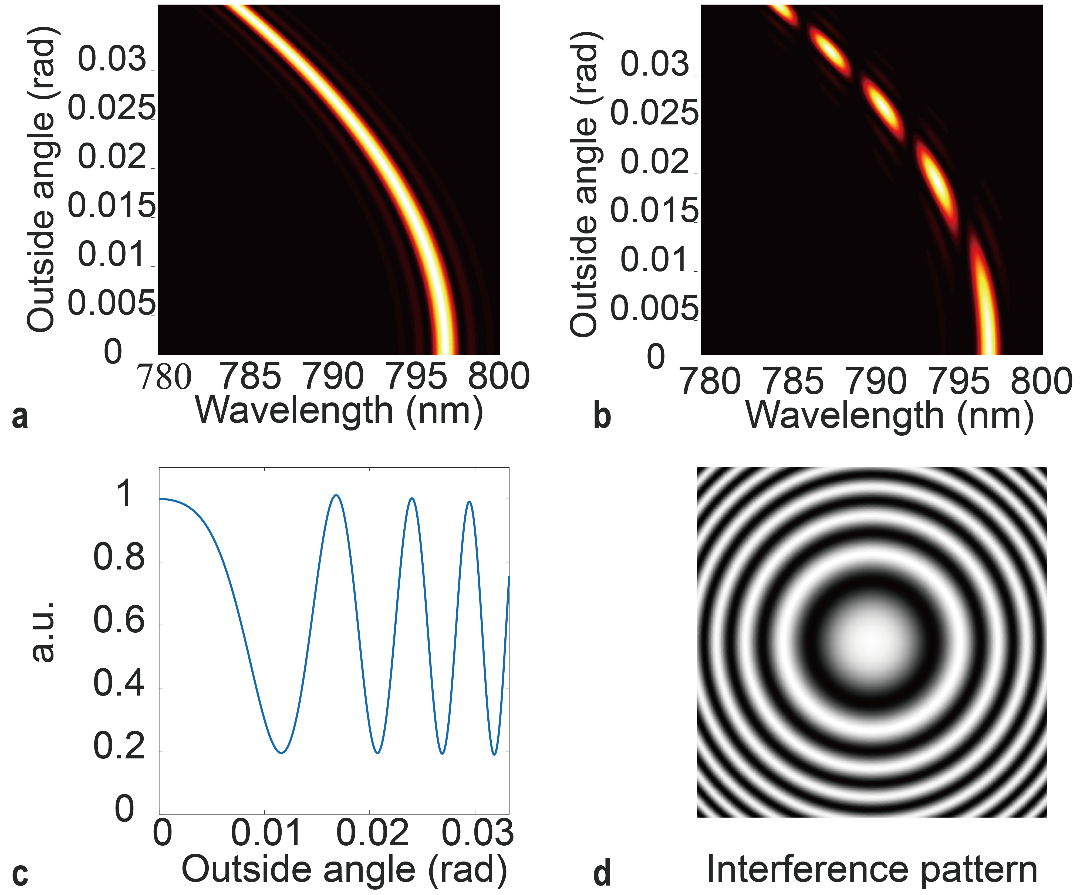


Fig. S2 | **a** /**b** The FAS before/after the interferometer. **c** The photon counts dependent on the outside angle. **d** Interference pattern with normalized intensity.

Then, we consider a Michelson interferometer shown in Fig. 2 in the main text. The divergent signal photons are collimated by a lens before the interferometer. When a monochromatic plane wave enters the interferometer, the output intensity should be multiplied by a factor $\left[1+\cos\left(4\pi d/\lambda_{\text{s}}\right)\right]/2$, where $d$ represents the arm difference of the Michelson interferometer and $2d$is the optical path difference. Therefore, the photon number after the interferometer becomes

$C_{2}\left(\lambda_{\text{s}},\theta_{\text{s\_out}}\right)=C_{1}\left(\lambda_{\text{s}},\theta_{\text{s\_out}}\right)\frac{1+\cos\left(4\pi d/\lambda_{\text{s}}\right)}{2}$ (S5)

The lenses before and after the interferometer form a 4-f imaging system. On the image plane, the FAS becomes the form shown in Fig. S2b (the same as that in Fig. 2b in the main text, there the FAS is an example with $d=100 \text{μm}$). The ICCD has almost equal response for different frequency components in a small band range. In the experiment, no narrowband filter is used expect for a 750 nm long-pass filter. By integrating over the wavelength, one can obtain the photon counts dependent on the angle

$\begin{matrix} C_{3}\left(\theta_{\text{s\_out}}\right)=\int_{-\infty}^{\infty} C_{2}\left(\lambda_{\text{s}},\theta_{\text{s\_out}}\right)\text{d}\lambda\\ =\frac{1}{2}\left[1+\alpha\int_{-\infty}^{\infty} \text{sinc}^{2}\left(\frac{\Delta k\cdot L}{2}\right)\cos\left(4\pi d/\lambda_{\text{s}}\right)\text{d}\lambda\right] \end{matrix}$ (S6)

Here, $C_{3}\left(\theta_{\text{s\_out}}\right)$ is a single-variable function of outside angle and the integral result shows the radial counts distribution and the example numerical integrating result is shown in Fig. S2c. Considering the rotational symmetry, ring-like interference patterns can be observed on the detection plane. Assume that $C\left(x,y\right)$ represent the counts on detection plane in Cartesian coordinate, then the interference pattern, shown in Fig. S2d, can be simulated using the relation $C\left(x,y\right)=C_{3}\left(\sqrt{x^{2}+y^{2}}/f_{3}\right)$, where the approximation $\theta_{\text{s\_out}}\approx\rho/f_{3}$ and $\rho=\sqrt{x^{2}+y^{2}}$are used and the integral in $C_{3}\left(\theta_{\text{s\_out}}\right)$ should be calculated numerically. In Section 4, Eq. (S21) can be seen as an approximate integrating result.

1. The approximate analytical expression for the tuning curve

In this section, we deduce the approximate parabolic expression for the tuning curve in some approximation. Firstly, we approximately regard the $\text{sinc}^{2}$ function in Eq. (2) as a delta function, this means $\Delta k=0$. Then, we give the lengths of three sides of the triangle in Fig. S1. Finally, the angle $\theta_{s}$ can be written directly using the cosine theorem.

We define some auxiliary quantities for convenience

$A=\frac{\omega_{\text{s}0}n_{\text{s}0}}{c}$ (S7)

$B=\frac{\omega_{\text{i}0}n_{\text{i}0}}{c}$ (S8)

$C=\frac{\omega_{\text{s}0}}{c}\left(\frac{dn}{d\omega_{\text{s}}}\right)_{\omega_{\text{s}0}}+\frac{n_{\text{s}0}}{c}=\frac{\beta_{\text{s}}\omega_{\text{s}0}+n_{\text{s}0}}{c}$ (S9)

$D=\frac{\omega_{\text{i}0}}{c}\left(\frac{dn}{d\omega_{\text{i}}}\right)_{\omega_{\text{i}0}}+\frac{n_{\text{i}0}}{c}=\frac{\beta_{\text{i}}\omega_{\text{i}0}+n_{\text{i}0}}{c}$ (S10)

where $\Delta\omega=\omega_{\text{s}}-\omega_{\text{s}0}=\omega_{\text{i}0}-\omega_{\text{i}}$; $\beta_{\text{s}}=\left(\frac{dn}{d\omega_{\text{s}}}\right)_{\omega_{\text{s}0}}, \beta_{\text{i}}=\left(\frac{dn}{d\omega_{\text{i}}}\right)_{\omega_{\text{i}0}}$are the first-order dispersion coefficient at the center frequency of signal and idler photons; $\omega_{\text{s}0}, \omega_{\text{i}0}, \lambda_{\text{s}0}, n_{\text{s}0}, n_{\text{i}0}$ are the center frequency and wavelength and the corresponding refractive index of signal and idler photons. Now the lengths of three sides of the triangle in Fig. S1b can be expressed as

$k_{\text{p}}-\frac{2\pi}{\Lambda}=\frac{n_{\text{s}0}\omega_{\text{s}0}+n_{\text{i}0}\omega_{\text{i}0}}{c}=A+B$ (S11)

$k_{\text{s}}=\frac{n\left(\omega_{\text{s}}\right)\omega_{\text{s}}}{c}\approx\left[n_{\text{s}0}+\left(\frac{dn}{d\omega_{\text{s}}}\right)_{\omega_{\text{s}0}}\Delta\omega\right]\frac{\left(\omega_{\text{s}0}+\Delta\omega\right)}{c}\approx A+C\Delta\omega$ (S12)

$k_{\text{i}}=\frac{n\left(\omega_{\text{i}}\right)\omega_{\text{i}}}{c}\approx\left[n_{\text{i}0}-\left(\frac{dn}{d\omega_{\text{i}}}\right)_{\omega_{\text{i}0}}\Delta\omega\right]\frac{\left(\omega_{\text{i}0}-\Delta\omega\right)}{c}\approx B-D\Delta\omega$ (S13)

The angle of signal photons in the crystal can be solved using the cosine theorem

$\begin{matrix} \cos\theta_{\text{s}}=\frac{-\left(B-D\Delta\omega\right)^{2}+\left(A+B\right)^{2}+\left(A+C\Delta\omega\right)^{2}}{2\left(A+B\right)\left(A+C\Delta\omega\right)} \\ \approx\frac{AB\text{+}A^{2}+\left(BD\text{+}AC\right)\Delta\omega}{AB\text{+}A^{2}+C\left(A+B\right)\Delta\omega} \\ \approx1-\frac{B\left(C-D\right)}{A\left(A+B\right)}\Delta\omega\end{matrix}$ (S14)

where the second-order terms including $\Delta\omega^{2}$are ignored and only the linear term including $\Delta\omega$ are preserved. Considering$\cos\theta_{\text{s}}\approx1-\frac{1}{2}\theta_{\text{s}}^{2}$, we have

$\theta_{\text{s}}^{2}=2\left[\frac{B\left(C-D\right)}{A\left(A+B\right)}\right]\Delta\omega=2\frac{\omega_{\text{i}0}n_{\text{i}0}}{\omega_{\text{s}0}n_{\text{s}0}}\frac{\beta_{\text{s}}\omega_{\text{s}0}+n_{\text{s}0}-\beta_{\text{i}}\omega_{\text{i}0}-n_{\text{i}0}}{\omega_{\text{s}0}n_{\text{s}0}+\omega_{\text{i}0}n_{\text{i}0}}\Delta\omega$ (S15)

Then, considering $\theta_{\text{s\_out}}\approx n_{\text{s}0}\theta_{\text{s}}$, we can obtain the outside angle

$\theta_{\text{s\_out}}^{2}=b_{1}\Delta\omega=-b_{2}\Delta\lambda$ (S16)

where $\Delta\lambda=\lambda_{\text{s}}-\lambda_{\text{s}0}$, $b_{2}=\frac{2\pi c}{\lambda_{\text{s}0}^{2}}b_{1}$, and

$b_{1}=\frac{2n_{\text{i}0}n_{\text{s}0}\omega_{\text{i}0}\left(\beta_{\text{s}}\omega_{\text{s}0}+n_{\text{s}0}-\beta_{\text{i}}\omega_{\text{i}0}-n_{\text{i}0}\right)}{\omega_{\text{s}0}\left(\omega_{\text{s}0}n_{\text{s}0}+\omega_{\text{i}0}n_{\text{i}0}\right)}$ (S17)

1. The interference visibility and the coherence length

The visibility is commonly dependent on the bandwidth. Here, the function $C_{1}\left(\lambda_{\text{s}},\theta_{\text{s\_out}}\right)$ in Eq. (S4) describes the spectrum for a particular outside angle and can be approximately written in another form

$C_{1}\left(\omega_{\text{s}},\theta_{\text{s\_out}}\right)=\frac{1}{\delta\omega_{\theta}}\text{sinc}^{2}\left[\frac{2\pi\left(\omega_{\text{s}}-\omega_{\theta}\right)}{\delta\omega_{\theta}}\right]$ (S18)

Where $\omega_{\theta}$ and $\delta\omega_{\theta}$ are the center frequency and the spectral full width of the FAS in Fig. S2a at the particular outside angle $\theta_{\text{s\_out}}$. Then, substituting Eq. (S18) into Eq. (5) and (6) , then the integrating becomes

$\begin{matrix} C_{3}\left(\theta_{\text{s\_out}}\right)=\frac{1}{2}\left[1+\int_{-\infty}^{\infty} \frac{1}{\delta\omega_{\theta}}\text{sinc}^{2}\left[\frac{2\pi\left(\omega_{\text{s}}-\omega_{\theta}\right)}{\delta\omega_{\theta}}\right]\cos\left(\frac{2d}{c}\omega_{\text{s}}\right)d\omega_{s}\right] \\ =\frac{1}{2}+\frac{1}{2}\text{tri}\left(\frac{\delta\omega_{\theta}}{2\pi c}d\right)\cos\left(\frac{2d\omega_{\theta}}{c}\right) \end{matrix}$ (S19)

where the triangle function is defined as

$\text{tri}\left(x\right)=\left\{\begin{matrix} 1-\left|x\right| , -1<x<1 \\ 0 , \text{otherwise} \end{matrix}\right.$ (S20)

Considering Eq. (S16), one can obtain $\omega_{\theta}=\theta_{\text{s\_out}}^{2}/b_{1}+\omega_{\text{s}0}$. The modified formula describing the interference becomes

$C\left(\overrightarrow{\rho};d\right)=\frac{1}{2}+\frac{1}{2}\text{tri}\left(\frac{\delta\omega_{0}}{2\pi c}d\right)\cos\left(\frac{2d\omega_{\text{s}0}}{c}\text{+}\frac{2d\rho^{2}}{cb_{1}f^{2}}\right)$ (S21)

where $\rho=f\theta_{\text{s\_out}}$ is the radial coordinate on the detection plane, $f$ is the focal length of the lens before detection plane. The bandwidth $\delta\omega_{\theta}$ has been approximately regarded as a constant $\delta\omega_{0}$ that is the acceptable bandwidth in the collinear case ($\theta_{\text{s\_out}}=0$). The visibility is given by

$V=\frac{C_{\max}-C_{\min}}{C_{\max}+C_{\min}}\approx\text{tri}\left(\frac{2d}{4\pi c/\delta\omega_{0}}\right)\approx\text{tri}\left(\frac{2d}{2\lambda_{\text{s}0}^{2}/\delta\lambda_{\text{s}0}}\right)$ (S22)

where $\delta\lambda_{\text{s}0}$ is the full linewidth of photons in the case of $\theta_{\text{s\_out}}=0$ and can be calculated by substituting Eq. (S1) into Eq. (S4). The triangle function describes the temporal coherence and this property can be simply reflected by a parameter, coherence length. Considering the full width of the triangle function, the coherence length is given by

$l_{\text{c}}=8\pi c/\delta\omega_{0}=4\lambda_{\text{s0}}^{2}/\delta\lambda_{\text{s0}}$ (S23)

The predicted coherence is $1.20 \text{mm}$ that agrees well with the experimental results shown in the main text. The linewidth is dependent on the material and length of the crystal. For a given material, the longer the crystal is the larger the coherence length is, the interference therefore requires a relatively long crystal to obtain visible stripes.

1. Determination of the equal path position of the Michelson interferometer


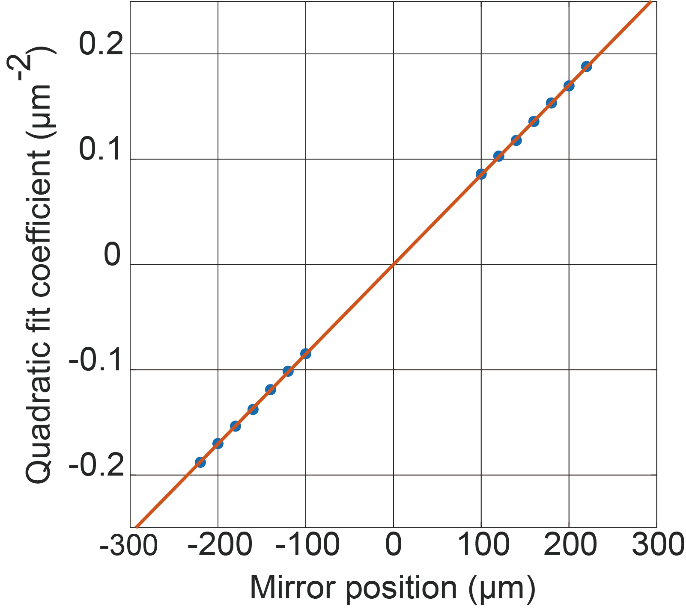


Fig. S3 | The quadratic coefficients $a$ are obtained by fitting the experimental data. The blue spots in the top right are the same as those in Fig. 3e in the main text; the blue spots in the bottom left are obtained by fitting the data recorded at $d=-\text{220，} -\text{200，} -\text{180，} -160, -140, -120\text{，} -100 \text{μm}$; the fitting is the same as that in Fig. 3d in the main text. The data (blue spots) are fitted by a linear function (red line).

In our experiment, the equal path position $d=0 \text{μm}$ in Fig. 3a~3c is set manually. Here, we determine the equal path position accurately by fitting the experimental data. In Fig. S3, blue spots show the quadratic coefficients at different position *d* of the mirror M2, which is fitted using a linear function $y=k\left(x-x_{0}\right)$. The fitting result shows the intercept $x_{0}$ at *x*-axis is $0.09\pm0.54 \text{μm}$, which is the real position of equivalent path. Considering the resolution of our displacement platform is $10 \text{μm}$, the error of $0.09 \text{μm}$ is negligible. The uncertainty $\pm0.54 \text{μm}$ is estimated using 95% confidence bounds of the fitting.

**Reference**

1 Boyd, R. W. Nonlinear Optics. 3th edn. (Amsterdam: Elsevier, 2008).

2 Hong, C. K. & Mandel, L. Theory of parametric frequency down conversion of light. *Phys Rev A* **31**, 2409-2418, doi:10.1103/physreva.31.2409 (1985).

3 Fradkin, K., Arie, A., Skliar, A. & Rosenman, G. Tunable midinfrared source by difference frequency generation in bulk periodically poled KTiOPO4. *Applied Physics Letters* **74**, 914 (1999).
